# Elevation of Tim-3 and PD-1 Expression on T Cells Appears Early in HIV Infection, and Differential Tim-3 and PD-1 Expression Patterns Can Be Induced by Common ***γ***-Chain Cytokines

**DOI:** 10.1155/2015/916936

**Published:** 2015-01-22

**Authors:** Zi-Ning Zhang, Ming-Lu Zhu, Yan-Hong Chen, Ya-Jing Fu, Tong-Wei Zhang, Yong-Jun Jiang, Zhen-Xing Chu, Hong Shang

**Affiliations:** ^1^Key Laboratory of AIDS Immunology of National Health and Family Planning Commission, Department of Laboratory Medicine, The First Affiliated Hospital, China Medical University, Shenyang 110001, China; ^2^Collaborative Innovation Center for Diagnosis and Treatment of Infectious Diseases, Hangzhou 310000, China; ^3^Department of Laboratory Medicine, Shenyang Fourth Hospital of the People, Shenyang 110031, China

## Abstract

*Purpose*. To explore the association between differential Tim-3 and PD-1 expression patterns and HIV disease progression, and to investigate the impact of common *γ*-chain cytokines on Tim-3 and PD-1 expression patterns on T cells. *Methods*. Tim-3/PD-1 expression on the T cells of patients with early and chronic HIV infections was detected. The expression levels and functional profiles of T cells with differential Tim-3 and PD-1 expression patterns induced by *γ*-chain cytokines were studied. *Results*. The elevation of differential Tim-3 and PD-1 expression patterns on T cells appeared early in HIV infection. Co-expression of Tim-3 and PD-1 (Tim-3+PD-1+) correlates with more severe exhaustion of T cells during HIV infection. In vitro stimulation of common *γ*-chain cytokines can induce differential expression patterns of Tim-3 and PD-1 on T cells. The enhancement of Tim-3 and PD-1 expression by common *γ*-chain IL-2 can inhibit the function of T cells re-stimulated by HIV gag and TCR, not by the re-stimulation of IL-2. *Conclusions*. The elevation of differential Tim-3 and PD-1 expression patterns on T cells represents a state of T cell exhaustion and can be induced by common *γ*-chain cytokines. These findings provide insights into HIV pathogenesis and help inform immune intervention strategies.

## 1. Introduction

Functional senescence of virus-specific T cells and the progressive loss of CD4+ T cells are features of HIV infection [1]. HIV triggers the overexpression of coinhibitory molecules on T cells, which contributes to a dysfunctional T-cell response with an “exhausted” phenotype [2]. PD-1 (programmed death-1) and Tim-3 (T-cell immunoglobulin mucin-3) are two major negative regulatory molecules associated with suboptimal T-cell responses in HIV infection, both in vitro and in vivo [3]. Although both molecules are involved in the regulation of T-cell exhaustion during chronic viral infection, Tim-3 and PD-1 belong to the Ig superfamily and the CD28/B7 family, respectively, and have been reported to be unique to certain populations of exhausted T cells in HIV infection [4]. There are three expression patterns of Tim-3 and PD-1 on T cells: Tim-3 and PD-1 coexpression (Tim-3+PD-1+) or Tim-3 and PD-1 individual expressions (Tim-3+PD-1− and Tim-3-PD-1+). Among T cells with these expression patterns, the Tim-3+PD-1+ T cells have been identified as the most exhausted phenotype in tumors and in mice with chronic viral infection [5–7]. Other T cells, such as Tim-3-PD-1+ or Tim-3+PD-1− T cells, exhibit different levels of exhaustion and ability to secrete cytokines [6, 8]. In HIV infection, Kassu et al. found a significant increase of HIV-specific CD4+ T cells expressing PD-1, CTLA-4, and TIM-3 in untreated subjects [9]. However, Jones et al. found that Tim-3 and PD-1 expression are found on distinct populations of T cells and tetramer+ HIV-1-specific CD8+ T cells were predominantly Tim-3+PD-1− [4]. Very little is known about the function of T cells with differential Tim-3 and PD-1 expression patterns or about their association with disease progression in HIV infection, especially during early HIV infection (EHI).

Common *γ*-chain (*γ*c)  cytokines, including interleukin- (IL-) 2, IL-4, IL-7, IL-9, IL-15, and IL-21, make up an important subfamily of the type I cytokinesthat regulate a variety of cellular responses, such as proliferation, differentiation, and survival [10]. IL-2, IL-7, and IL-15 are primary regulators of T-cell homeostasis and thus have been considered prime immunotherapeutic candidates in HIV infection, both for increasing T-cell levels/function and for augmenting vaccine-elicited viral-specific T-cell responses [11]. Due to the pleiotropy and redundancy of cytokines [12], it is vital to have a comprehensive knowledge of the role of *γ*c cytokines in the regulation of T-cell function. Studies have shown that *γ*c cytokines induce the expression of Tim-3 or PD-1 expression [13, 14], but the induction of the comprehensive expression pattern of Tim-3 and PD-1 by *γ*c cytokines was less clear. Given that differential expression patterns of Tim-3 and PD-1 may have distinct functions, further study of the sensitivity of Tim-3 and PD-1 expression patterns to *γ*c cytokines may provide useful information on how *γ*c cytokines regulate immunity.

In this study, we found that the elevation of differential Tim-3 and PD-1 expression patterns on T cells appears early in HIV infection. Coexpression of Tim-3 and PD-1 (Tim-3+PD-1+) correlates with more severe exhaustion of T cells during HIV infection, and the simultaneous blockade of Tim-3 and PD-1 pathways synergistically restores T-cell secretion of IFN-*γ*/IL-2. In vitro stimulation of common *γ*c cytokines mediated the induction of Tim-3 and PD-1 expression patterns, and the enhancement of Tim-3 and PD-1 expression by common *γ*c cytokine IL-2 can inhibit the function of T cells restimulated by HIV gag and TCR, not by the restimulation of IL-2.

## 2. Materials and Methods

### 2.1. Subjects

This study enrolled 44 treatment-naïve HIV-infected patients, including nine patients with early HIV infection (EHI, 9 males) and 35 patients with chronic HIV infection (CHI, 34 males and 1 female). EHI was defined as documented HIV-1 acquisition within the previous one year [15], and CHI was defined as HIV-1 infection for more than two years. Clinical data obtained from the patients in this study were the following (Mean ± SD): EHI, absolute CD4+ T-cell counts = 470 ± 182 cells/*μ*L, and Log10 viral loads = 4.75 ± 0.64 copies/mL; CHI, absolute CD4+ T-cell counts = 368 ± 177 cells/*μ*L, and Log10 viral loads = 3.97 ± 0.78 copies/mL. For normal controls (NC), nine individuals were included from the same demographic area and age range as the study subjects. Ethical approval for this study was obtained from the local ethical review committee, and written informed consent for participation in the study was obtained from all patients.

### 2.2. Detection of Tim-3 and PD-1 Expression on Primary and *γ*c Cytokine-Stimulated PBMCs

Peripheral blood mononuclear cells (PBMCs) were isolated by Ficoll gradient (Sigma) and were washed once with FACS buffer (PBS, with 1% bovine serum albumin). For staining, 1 × 10^6^ cells were incubated with conjugated antibodies APC-cy7-conjugated anti-CD3, PerCP-conjugated anti-CD8 (BD Biosciences Pharmingen), PE-conjugated anti-Tim-3, and FITC-conjugated anti-PD-1 (Biolegend) for 30 min at 4°C. Analysis was performed using an LSRII instrument (BD Biosciences), and at least 10,000 events were collected. Tim-3 and PD-1 expression on CD4+ (defined as CD3+CD8− cells) and CD8+ T cells were analyzed with FACSDiva software (BD Biosciences). For the detection of cytokine-induced Tim-3 and PD-1 expression, freshly isolated PBMCs were plated in round-bottom 96-microtiter plates at 0.5 million cells/well in 200 *μ*L complete RPMI 1640 containing anti-CD3 (5 *μ*g/mL) and anti-CD28 (2 *μ*g/mL) antibodies (BD Biosciences) or recombinant IL-2, IL-7, IL-15, and IL-21 (25 ng/mL) (IL-2, IL-7, and IL-15: R&D Systems; IL-21: Biosource). Cells were cultured for five days and were incubated with conjugated antibodies against PerCP-conjugated anti-CD3, APC-cy7-conjugated anti-CD4 (BD Biosciences Pharmingen), PE-conjugated anti-Tim-3, and FITC-conjugated anti-PD-1 for 30 min at 4°C. Analysis was performed using an LSRII instrument and analyzed with FlowJo software.

### 2.3. TCR and Antigen-Specific Stimulation and Intracellular Staining

PBMCs were plated in round-bottom 96-microtiter plates at 500,000 cells/well in 200 *μ*L complete RPMI 1640 containing anti-CD3 (5 *μ*g/mL) and anti-CD28 (2 *μ*g/mL) antibodies (BD Biosciences) or HIV gag peptide (10 *μ*g/mL, XiAn Meilian Company), with or without anti-Tim-3 and anti-PD-1 (25 *μ*g/mL) antibodies (Biolegend). Cells were cultured for three days, and GolgiStop was added during the last five hours of the culture. The cells were incubated with conjugated antibodies PerCP-conjugated anti-CD3, APC-cy7-conjugated anti-CD4, PE-conjugated anti-Tim-3, and FITC-conjugated anti-PD-1, and intracellular cytokine staining for APC-conjugated anti-IFN-*γ* (BD Biosciences Pharmingen) or APC-conjugated IL-2 (Biolegend) was carried out using the Cytofix/Cytoperm Fixation/Permeabilization Kit according to the manufacturer's instructions (BD Biosciences). Intracellular expression of IL-2 or IFN-*γ* within CD4+ and CD8+ (CD3+CD4−) T cells was detected using an LSRII instrument and analyzed with FlowJo software.

### 2.4. Cytokine Stimulation and Intracellular Staining

PBMCs were initially stimulated with IL-2 (100 U/mL) for six days. Cells were then washed and restimulated with plate-bound anti-CD3/CD28 antibodies (anti-CD3, 0.2 *μ*g/mL; anti-CD28, 0.4 *μ*g/mL), IL-2 (100 U/mL), or HIV gag peptide (10 *μ*g/mL), with or without anti-Tim-3/PD-1 antibodies (25 *μ*g/mL). For the detection of IL-2 and IFN-*γ* production, GolgiStop was added 1 hour after restimulation, and 4 hours later cells were stained for PerCP-conjugated anti-CD3 and APC-cy7-conjugated anti-CD4 and intracellular APC-conjugated anti-IL-2 or APC-conjugated anti-IFN-*γ*, according to the manufacturer's recommendations (BD Biosciences). Intracellular expression of IL-2 or IFN-*γ* within CD4+ and CD8+ (CD3+CD4−) T cells was detected using an LSRII instrument and analyzed with FlowJo software.

### 2.5. Statistical Analysis

SPSS 17.0 software (SPSS Inc.) was used to conduct statistical analyses. Mann-Whitney* U* tests were used to assess differences between the variables of different groups. Correlations between the variables were evaluated using the Spearman rank correlation test. Comparisons of IFN-*γ* and IL-2 secretion before and after the anti-PD-1/Tim-3 blockade as well as Tim-3 and PD-1 expression on T cells before and after *γ*c cytokine-stimulation were performed by the Wilcoxon matched-pairs* t* test. *P* values less than 0.05 were considered to be statistically significant.

## 3. Results

### 3.1. Elevation of Tim-3 and PD-1 Expression on T Cells Appears Early in HIV Infection and Correlates with Disease Progression

**Figure 1:**
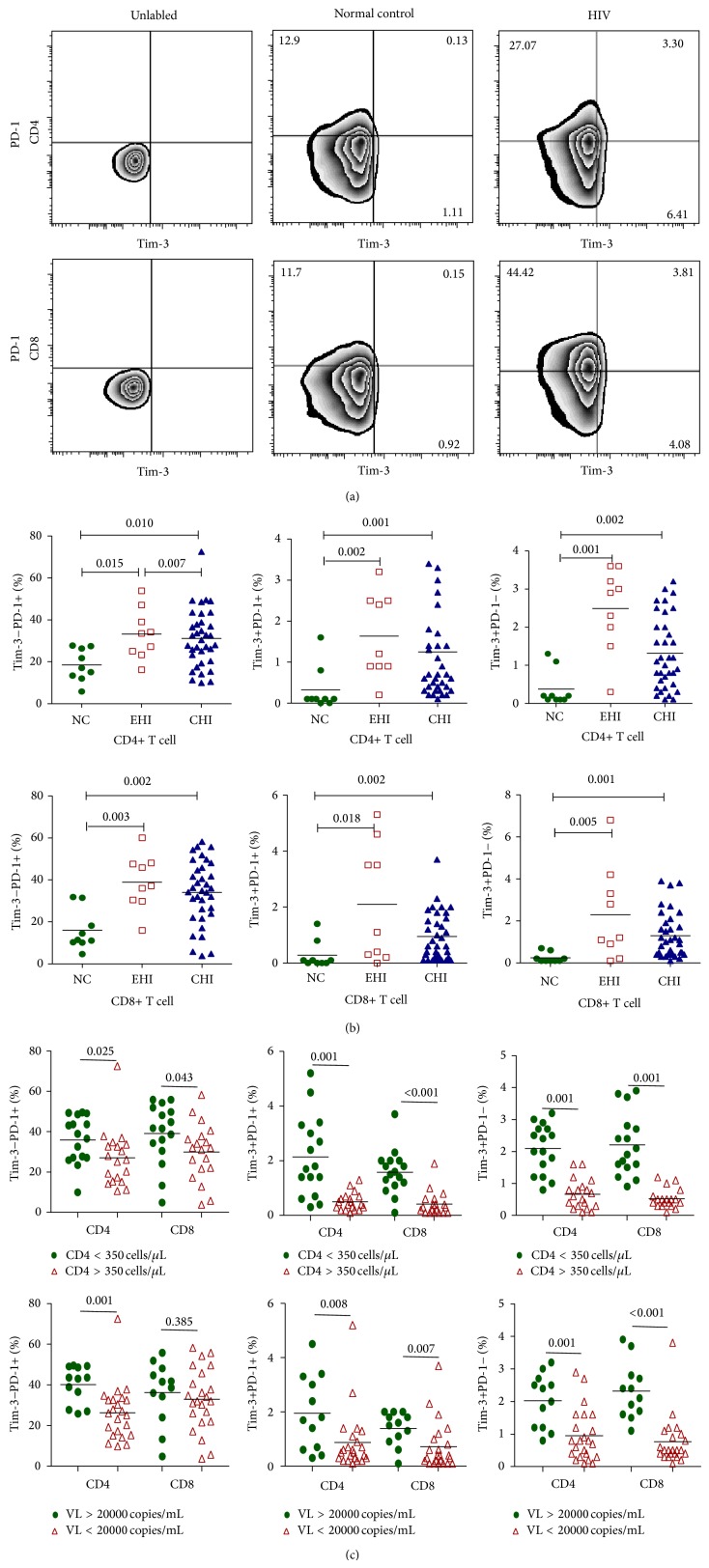
Comparison of levels of differential expression patterns of Tim-3 and PD-1 (Tim-3+PD-1+, Tim-3-PD-1+, and Tim-3+PD-1−) on T cells in HIV-1 infected patients and normal controls. (a) Representative data regarding the expression patterns of Tim-3 and PD-1 in a normal control and in a chronic HIV-infected patient. (b) The percentages of differential Tim-3/PD-1 expression patterns on the CD4+ and CD8+ T cells of normal controls (NC, *n* = 9), early HIV-infected patients (EHI, *n* = 9), and chronic HIV-infected patients (CHI, *n* = 35). (c) The percentages of Tim-3/PD-1 expression patterns on the CD4+ and CD8+ T cells of chronic HIV-infected patients with different CD4+ T-cell counts (more than or less than 350 cells/*μ*L) and viral loads (more than or less than 20,000 copies/mL).

Among the three expression patterns of Tim-3 and PD-1 on T cells, we found that the frequencies of Tim-3-PD-1+, Tim-3+PD-1−, and Tim-3+PD-1+ expressions on CD4+ and CD8+ T cells were all significantly higher in individuals with chronic HIV infections than in the normal controls (CD4+Tim-3-PD-1+: NC, 18.48 ± 7.74; CHI, 31.09 ± 13.67; CD4+Tim-3+PD-1+: NC, 0.32 ± 0.54; CHI, 1.25 ± 1.29; CD4+Tim-3+PD-1−: NC, 0.38 ± 0.47; CHI, 1.32 ± 0.94; CD8+Tim-3-PD-1+: NC, 15.92 ± 9.60; CHI, 34.06 ± 15.02; CD8+Tim-3+PD-1+: NC, 0.28 ± 0.49; CHI, 0.95 ± 0.86; CD8+Tim-3+PD-1−: NC, 0.24 ± 0.24; CHI, 1.30 ± 1.08) (*P* < 0.05, Figures 1(a) and 1(b)). To see whether Tim-3 and PD-1 expression patterns on T cells differed with the severity of the HIV infection, we divided the CHIs into two groups according to their CD4+ T-cell counts and viral loads (VLs). We found that the levels of Tim-3-PD-1+, Tim-3+PD-1−, and Tim-3+PD-1+ expression on T cells were all significantly higher in patients with severe infections (CD4+ T cells < 350 cells/*μ*L, or VL > 20,000 copies/mL) than in patients whose infections were less severe (CD4+ T cells > 350 cells/*μ*L, or VL < 20,000 copies/mL) (*P* < 0.05, except for CD8+Tim-3-PD-1+ T cells, Figure 1(c)).

**Figure 2:**
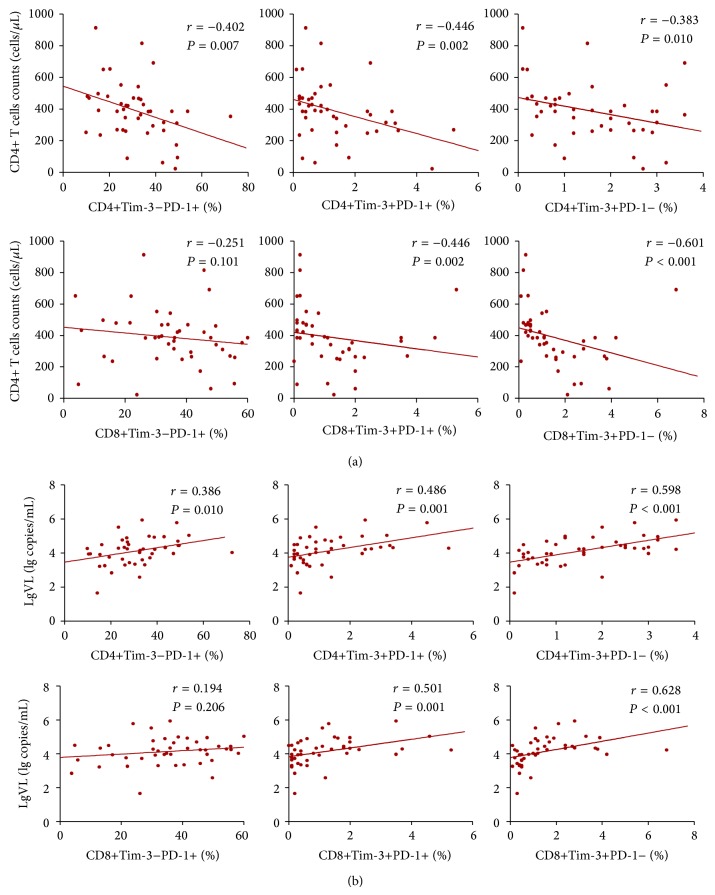
Correlation between percentages of differential Tim-3/PD-1 expression patterns (Tim-3-PD-1+, Tim-3+PD-1+, and Tim-3+PD-1−) on CD4+ and CD8+ T cells and (a) CD4+ T-cell counts and (b) viral loads.

Through the study of EHIs, we found that the elevation of Tim-3 and PD-1 expression on T cells occurs in early HIV infection. The frequencies of Tim-3+PD-1+, Tim-3+PD-1−, and Tim-3-PD-1+ expressions on CD4+ and CD8+ T cells were significantly higher in EHIs than that in normal controls (in EHIs: CD4+Tim-3-PD-1+, 33.22 ± 11.95; CD4+Tim-3+PD-1+, 1.63 ± 1.02; CD4+Tim-3+PD-1−, 2.48 ± 1.09; CD8+Tim-3-PD-1+, 38.94 ± 12.97; CD8+Tim-3+PD-1+, 2.10 ± 2.11; CD8+Tim-3+PD-1−, 2.28 ± 2.21) (*P* < 0.05, Figure 1(b)). We then studied the relationship between Tim-3/PD-1 expression patterns and CD4+ T-cell counts and VLs of all 44 HIV-infected patients, including EHIs and CHIs. We found that the expression levels of Tim-3-PD-1+, Tim-3+PD-1−, and Tim-3+PD-1+ on CD4+ and CD8+ T cells correlated negatively with CD4+ T-cell counts (Figure 2(a)) and correlated positively with viral loads (Figure 2(b), *P* < 0.05), except for CD8+Tim-3-PD-1+ T cells.

### 3.2. Coexpression of Tim-3 and PD-1 Correlates with More Severe Exhaustion of T Cells during HIV Infection, and Simultaneous Blockade of Tim-3 and PD-1 Pathways Synergistically Restores T-Cell Secretion of IFN-*γ* or IL-2

**Figure 3:**
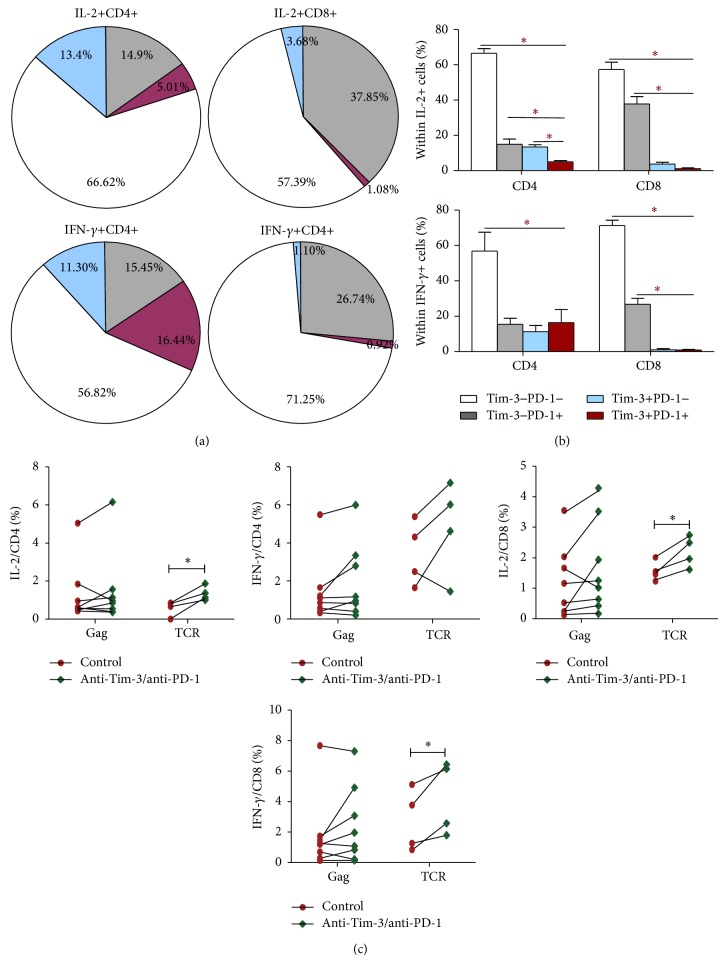
Functional study of Tim-3/PD-1 expression patterns on T cells. (a) PBMCs from HIV-infected patients (*n* = 4) were stimulated with TCR (anti-CD3/CD28) for 3 days, and IL-2 and IFN-*γ* production by T cells were detected. Distributions of Tim-3/PD-1 expression patterns on IFN-*γ*- and IL-2-producing T cells are indicated. (b) Statistical analysis of the distribution of differential Tim-3/PD-1 expression patterns on the IFN-*γ*- and IL-2-producing T cells (^*^ *P* < 0.05). (c) PBMCs from HIV-infected patients were stimulated with anti-CD3 and anti-CD28 (*n* = 4) antibodies or HIV gag peptides (*n* = 8) with or without blocking antibodies (anti-Tim-3 and PD-1). IFN-*γ* and IL-2 production by CD4+ and CD8+ T cells before and after the anti-Tim-3/PD-1 blockade were compared (^*^ *P* < 0.05).

We next studied whether the differential expression patterns of Tim-3 and PD-1 resulted in functional differences. We stimulated PBMCs with TCR antibodies (anti-CD3/CD28) to compare the IL-2 and IFN-*γ* productions of T cells with differential Tim-3 and PD-1 expression patterns. We found that, among IFN-*γ*- and IL-2-producing T cells, Tim-3-PD-1− T cells were the greatest in number, followed by Tim-3-PD-1+, then by Tim-3+PD-1−, and then by Tim-3+PD-1+ T cells (Figure 3(a), except for CD4+IFN-*γ*+ T cells). The proportion of the Tim-3+PD-1+ subset within IFN-*γ*/IL-2-secreting CD4+ and CD8+ T cells was lower than that of the Tim-3-PD-1- and Tim-3-PD-1+ subsets, except for IFN-*γ*-secreting CD4+ T cells (*P* < 0.05) (Figure 3(b)).

In a next round of experiments, we simultaneously blocked Tim-3 and PD-1 to investigate whether this blockade could restore IFN-*γ*/IL-2 secretion from T cells during chronic HIV infection. PBMCs from chronic HIV-infected patients were stimulated with anti-CD3 and anti-CD28 antibodies (*n* = 4) or HIV gag peptides (*n* = 8), with or without blocking antibodies (anti-Tim-3 and anti-PD-1). The levels of IFN-*γ* and IL-2 secretion by CD4+ and CD8+ T cells were detected. We found a significant increase in the levels of IL-2 and IFN-secretion by CD4+ and CD8+ T cells (*P* < 0.05) that were stimulated with anti-CD3/CD28 in the presence of anti-Tim-3/PD-1 mAbs, as compared to cells that were not blocked (Figure 3(c)). T-cell production of IL-2 and IFN-*γ* also showed a tendency to increase after stimulation by gag peptide with a dual blockade compared to the cells that were not blocked, albeit the difference was not statistically significant (Figure 3(c)).

### 3.3. In Vitro Common *γ*c Cytokine-Mediated Induction of Tim-3 and PD-1 Expression Patterns and Its Functional Consequences

**Figure 4:**
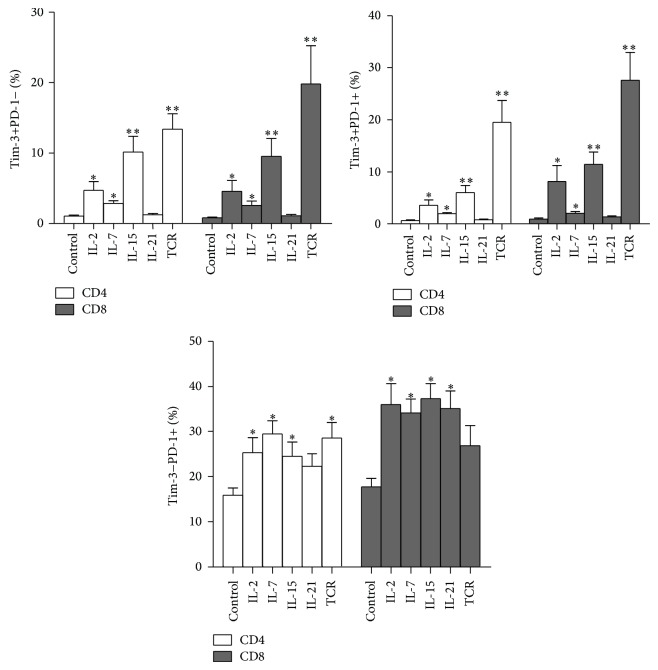
Common *γ*c cytokine-mediated induction of Tim-3/PD-1 expression patterns on T cells in PBMCs from HIV patients. Tim-3 and PD-1 expression on T cells were analyzed following a five-day period of stimulation and compared with those of cells cultured in control medium (*n* = 12). This figure compares the abilities of different common *γ*c cytokines to induce the expression of the Tim-3+PD-1+, Tim-3-PD-1+ and Tim-3+PD-1− patterns on T cells (^*^ *P* < 0.05; ^**^ *P* < 0.01).

To further investigate alterations of Tim-3 and PD-1 expression patterns induced by cytokine stimulation, we stimulated PBMCs from HIV patients (*n* = 12) with *γ*c cytokines, including IL-2, IL-7, IL-15, and IL-21 for five days. We found that common *γ*c cytokines IL-2, IL-7, and IL-15 can significantly elevate the expression of Tim-3+PD-1+, Tim-3+PD-1−, and Tim-3-PD-1+ subsets on T cells. IL-21 can increase the expression of Tim-3-PD-1+ subsets but seems to have no effect on the expression patterns of Tim-3+PD-1+ and Tim-3+PD-1− on T cells (Figure 4).

**Figure 5:**
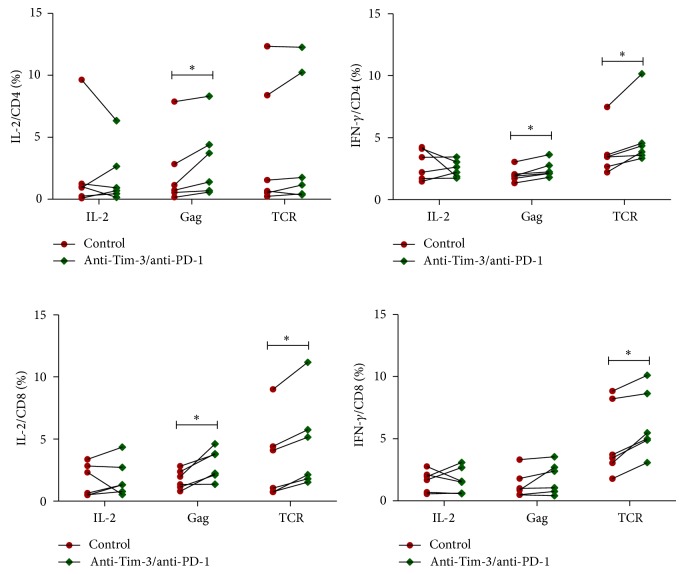
Functional consequences of *γ*c cytokine-mediated modulation of Tim-3 and PD-1 expression on T cells. PBMCs prestimulated with IL-2 (*n* = 5) were harvested at 6 days after stimulation, washed, and then restimulated with HIV gag peptides, TCR, or IL-2 in the presence of blocking antibodies (anti-Tim-3/PD-1). IL-2 and IFN-*γ* production by CD4+ and CD8+T cells were detected by intracellular cytokine staining.

Since *γ*c cytokines trigger an increase in the expression of Tim-3 and PD-1 on T cells, we performed experiments to observe the functional consequences of this alteration. PBMCs that were prestimulated with IL-2 (*n* = 5) were harvested at 6 days after stimulation, washed, and then restimulated with IL-2, HIV gag peptides, or TCR in the presence of blocking antibodies (anti-Tim-3 and anti-PD-1). IL-2 and IFN-*γ* secretions by T cells were detected by intracellular cytokine staining after 5 hours of stimulation. We found that, after 6 days of IL-2 stimulation, anti-Tim-3/PD-1 antibodies can restore gag- and TCR-stimulated IL-2 or IFN-*γ* production by CD4+ and CD8+ T cells (*P* < 0.05, except for gag-stimulated IFN-*γ* production by CD8+ T cells and TCR-stimulated IL-2 production by CD4+ T cells, Figure 5). However, dual blockade of Tim-3 and PD-1 did not enhance cytokine production by T cells after IL-2 restimulation (Figure 5). These results suggest that the enhancement of PD-1 and Tim-3 expression by *γ*c cytokine IL-2 can inhibit function of T cells stimulated by HIV gag and TCR, but this enhancement cannot inhibit the T-cell function restimulated by IL-2.

## 4. Discussion

Both coinhibitory molecules and common *γ*c cytokines have strong potential for inclusion in the development of therapeutic interventions that augment the functionality of host immune cells, which would lead to improved immune control of HIV infection. It is worth investigating the dynamics of Tim-3/PD-1 expression patterns on T cells in HIV disease progression and the induction of their expression by common *γ*c cytokines. Findings from this investigation could provide insights into HIV pathogenesis and help inform immune intervention strategies. In this study, we investigated the association between differential Tim-3 and PD-1 expression patterns (coexpression or individual expression) and HIV disease progression, as well as the impact of *γ*c cytokines on Tim-3 and PD-1 expression patterns on T cells.

While antiretroviral therapy (ART) regimens have proven to be effective in controlling active HIV replication, complete recovery of CD4+ T-cell counts does not always occur, even among patients who display high levels of virologic control [11]. Different adjuvant therapies, including immunomodulation, are being tested in clinical trials or are under consideration in hopes of addressing these remaining challenges [2]. Over the past few years, Tim-3 and PD-1 have emerged as attractive potential targets in therapy developments because not only are they responsible for HIV-specific T-cell impairment, but also they play a wider role in HIV pathogenesis [2]. The dynamics of PD-1 and Tim-3 when individually expressed on T cells have been well studied [4, 16], but the effect of differential expression patterns of Tim-3 and PD-1 in HIV infection is not clear. In this study, we found that the number of T cells with various Tim-3 and PD-1 expression patterns (Tim-3+PD-1+, Tim-3-PD-1+, and Tim-3+PD-1−) was elevated in chronic HIV infection and that expression levels significantly correlated with disease progression. We have stated that, in early HIV infection, the elevation of all expression patterns of Tim-3 and PD-1 is present and that expression levels were not significantly different compared to expression levels in chronic HIV infection, indicating that T-cell exhaustion occurs in early HIV infection. Therefore, treatment of early HIV infection could involve the alteration of coinhibitory molecules such as Tim-3 and PD-1.

Given the known effects of *γ*c cytokines on the growth, differentiation, and survival of T cells, their therapeutic use alongside HAART may further improve immunity reconstitution in infected patients [17, 18]. Recently, two reports about a “functional cure” in an HIV-infected infant and in 14 patients who were treated within the first 2 months of infection highlight the importance of early treatment for increasing the chances of affecting a functional HIV cure [19, 20]. It is worth investigating whether current and emerging cytokine therapies could provide supplementary reinforcement to current pharmaceutical treatments in early infection. In this study, we found that differential expression patterns of Tim-3 and PD-1 on T cells were associated with decreased T-cell function, such as decreased cytokine production, especially the Tim-3+PD-1+ and Tim-3+PD-1− expression patterns. Our follow-up experiments showed that most *γ*c cytokines can significantly increase the number of Tim-3+PD-1+ and Tim-3+PD-1− cells. Our results suggest that applying *γ*c cytokines in a clinical setting should be done with caution because they can increase the generation of exhausted T cells. Because the various expression patterns of Tim-3 and PD-1 are elevated early in HIV infection, the use of cytokines as reinforcements for ART in early infection may aggravate the exhaustion of T cells. Previous studies revealed that IL-21 may have a positive effect on T, B, and NK cells [21–28] without an associated increase in cellular proliferation or activation [22, 25, 27, 29]. In our study, we found that IL-21 can increase the expression of Tim-3-PD-1+ on T cells. Kinter et al. also found that IL-21 can upregulate PD-1 expression on purified T cells [13]. We found that IL-21 did not increase Tim-3+PD-1+ and Tim-3+PD-1− expression patterns. Mujib et al. found that IL-21 increased Tim-3 expression on T cells, but to a lesser extent compared with the increase effect by IL-2, IL-7, and IL-15 [14]. Our finding suggests that IL-21 could be a better candidate for use in immunotherapeutic approaches in established SIV/HIV infection as it has the lesser extent of affecting factors that favor disease progression, such as coinhibitory molecules and activation [30].

In our study, we found that, after 6 days of IL-2 stimulation, dual blockade of Tim-3 and PD-1 can restore gag- and TCR-stimulated IL-2 or IFN-*γ* production by T cells but does not restore IL-2 or IFN-*γ* production of T cells by restimulation with IL-2. We focused on IL-2 because it is the most studied cytokine among the common *γ*c cytokines in the clinical treatment of HIV infection. Study of the functional consequences of IL-2-induced PD-1 and Tim-3 expression may provide more useful information in clinical application than the study of other cytokines, such as IL-15, which also modulated the expression of Tim-3 and PD-1 on CD4+ and CD8+ T cells in our study. Kinter et al. also found that PD-1 enhancement by *γ*c cytokines does not inhibit *γ*c cytokine-induced proliferation or signaling events, which may be because PD-1 engagement requires TCR-triggered SHP-2 recruitment to the cytoplasmic tail of PD-1. Therefore, without TCR, PD-1 ligation by itself does not generate a suppressive signal to *γ*c cytokine-induced proliferation or signaling events [13]. Although *γ*c cytokines can also independently induce Tim-3 expression [14], the consequences of cytokine-induced Tim-3 and PD-1 coexpression had not been previously reported. In our study, we showed that the simultaneous blockade of IL-2-induced Tim-3 and PD-1 expression restored the function of T cells stimulated by HIV gag and TCR but cannot restore the function of T cells restimulated by IL-2. Tim-3 and PD-1 have an additive and sometimes synergistic effect on the invigoration of exhausted T cells [31]. PD-1 inhibits the induction of phosphatidylinositol-3-kinase (PI3K) activity as well as the downstream activation of Akt [32]. Unlike other negative regulators of T-cell function (e.g., PD-1), Tim-3 does not contain any obvious inhibitory signaling motifs, and its downstream signaling targets remain unknown [4]. Lee et al. found that Tim-3 can directly bind to the p85 PI3K adaptor [31], indicating that the inhibition of PI3K and Akt may be involved in both the Tim-3 and PD-1 functions. This involvement may explain the observed different response of Tim-3 and PD-1 coexpression to IL-2 and TCR restimulation. IL-2 can activate Akt through STAT5 [33], which may circumvent PD-1- and Tim-3-mediated inhibition of PI3K-Akt activation.

In summary, we found that the elevation of Tim-3 and PD-1 expression on T cells appears early in HIV infection and that differential Tim-3 and PD-1 expression patterns can be induced by common *γ*c cytokines. PD-1 and Tim-3 expression by *γ*c cytokines can inhibit the cytokine secretion stimulated by HIV gag and TCR. Our findings could inform ongoing investigations regarding HIV pathogenesis and could contribute to the development of immune intervention strategies by interpreting the roles for coinhibitory molecules and cytokines in the treatment of HIV infection.

## References

[1] Boliar S., Murphy M. K., Tran T. C. (2012). B-lymphocyte dysfunction in chronic HIV-1 infection does not prevent cross-clade neutralization breadth. *Journal of Virology*.

[2] Porichis F., Kaufmann D. E. (2012). Role of PD-1 in HIV pathogenesis and as target for therapy. *Current HIV/AIDS Reports*.

[3] Larsson M., Shankar E. M., Che K. F. (2013). Molecular signatures of T-cell inhibition in HIV-1 infection. *Retrovirology*.

[4] Jones R. B., Ndhlovu L. C., Barbour J. D. (2008). Tim-3 expression defines a novel population of dysfunctional T cells with highly elevated frequencies in progressive HIV-1 infection. *Journal of Experimental Medicine*.

[5] Fourcade J., Sun Z., Benallaoua M. (2010). Upregulation of Tim-3 and PD-1 expression is associated with tumor antigen-specific CD8^+^ T cell dysfunction in melanoma patients. *The Journal of Experimental Medicine*.

[6] Sakuishi K., Apetoh L., Sullivan J. M., Blazar B. R., Kuchroo V. K., Anderson A. C. (2010). Targeting Tim-3 and PD-1 pathways to reverse T cell exhaustion and restore anti-tumor immunity. *Journal of Experimental Medicine*.

[7] Jin H.-T., Anderson A. C., Tan W. G. (2010). Cooperation of Tim-3 and PD-1 in CD8 T-cell exhaustion during chronic viral infection. *Proceedings of the National Academy of Sciences of the United States of America*.

[8] Zhou Q., Munger M. E., Veenstra R. G. (2011). Coexpression of Tim-3 and PD-1 identifies a CD8^+^ T-cell exhaustion phenotype in mice with disseminated acute myelogenous leukemia. *Blood*.

[9] Kassu A., Marcus R. A., D'Souza M. B. (2010). Regulation of virus-specific CD4^+^ T cell function by multiple costimulatory receptors during chronic HIV infection. *Journal of Immunology*.

[10] Yamane H., Paul W. E. (2012). Cytokines of the *γ*
_c_ family control CD4^+^ T cell differentiation and function. *Nature Immunology*.

[11] Leone A., Picker L. J., Sodora D. L. (2009). IL-2, IL-7 and IL-15 as immuno-modulators during SIV/HIV vaccination and treatment. *Current HIV Research*.

[12] Ozaki K., Leonard W. J. (2002). Cytokine and cytokine receptor pleiotropy and redundancy. *The Journal of Biological Chemistry*.

[13] Kinter A. L., Godbout E. J., McNally J. P. (2008). The common *γ*-chain cytokines IL-2, IL-7, IL-15, and IL-21 induce the expression of programmed death-1 and its ligands. *The Journal of Immunology*.

[14] Mujib S., Jones R. B., Lo C. (2012). Antigen-independent induction of tim-3 expression on human T cells by the common *γ*-chain cytokines IL-2, IL-7, IL-15, and IL-21 is associated with proliferation and is dependent on the phosphoinositide 3-kinase pathway. *The Journal of Immunology*.

[15] Streeck H., Jolin J. S., Qi Y. (2009). Human immunodeficiency virus type 1-specific CD8^+^ T-cell responses during primary infection are major determinants of the viral set point and loss of CD4^+^ T cells. *Journal of Virology*.

[16] Day C. L., Kaufmann D. E., Kiepiela P. (2006). PD-1 expression on HIV-specific T cells is associated with T-cell exhaustion and disease progression. *Nature*.

[17] Sirskyj D., Thèze J., Kumar A., Kryworuchko M. (2008). Disruption of the *γ*c cytokine network in T cells during HIV infection. *Cytokine*.

[18] Sereti I., Estes J. D., Thompson W. L. (2014). Decreases in colonic and systemic inflammation in chronic HIV infection after IL-7 administration. *PLoS Pathogens*.

[19] Persaud D., Gay H., Ziemniak C. (2013). Absence of detectable HIV-1 viremia after treatment cessation in an infant. *The New England Journal of Medicine*.

[20] Sáez-Cirión A., Bacchus C., Hocqueloux L. (2013). Post-treatment HIV-1 controllers with a long-term virological remission after the interruption of early initiated antiretroviral therapy ANRS VISCONTI Study. *PLoS Pathogens*.

[21] Yue F. Y., Lo C., Sakhdari A. (2010). HIV-specific IL-21 producing CD4+ T cells are induced in acute and chronic progressive HIV infection and are associated with relative viral control. *The Journal of Immunology*.

[22] Iannello A., Boulassel M.-R., Samarani S. (2010). IL-21 enhances NK cell functions and survival in healthy and HIV-infected patients with minimal stimulation of viral replication. *Journal of Leukocyte Biology*.

[23] Iannello A., Boulassel M.-R., Samarani S. (2010). Dynamics and consequences of IL-21 production in HIV-infected individuals: a longitudinal and cross-sectional study. *Journal of Immunology*.

[24] Hogg A. E., Bowick G. C., Herzog N. K., Cloyd M. W., Endsley J. J. (2009). Induction of granulysin in CD8^+^ T cells by IL-21 and IL-15 is suppressed by human immunodeficiency virus-1. *Journal of Leukocyte Biology*.

[25] Strbo N., de Armas L., Liu H., Kolber M. A., Lichtenheld M., Pahwa S. (2008). IL-21 augments natural killer effector functions in chronically HIV-infected individuals. *AIDS*.

[26] Iannello A., Tremblay C., Routy J.-P., Boulassel M.-R., Toma E., Ahmad A. (2008). Decreased levels of circulating IL-21 in HIV-infected AIDS patients: correlation with CD4^+^ T-cell counts. *Viral Immunology*.

[27] White L., Krishnan S., Strbo N. (2007). Differential effects of IL-21 and IL-15 on perforin expression, lysosomal degranulation, and proliferation in CD8 T cells of patients with human immunodeficiency virus-1 (HIV). *Blood*.

[28] Bolesta E., Kowalczyk A., Wierzbicki A. (2006). Increased level and longevity of protective immune responses induced by DNA vaccine expressing the HIV-1 Env glycoprotein when combined with IL-21 and IL-15 gene delivery. *Journal of Immunology*.

[29] Parmigiani A., Pallin M. F., Schmidtmayerova H., Lichtenheld M. G., Pahwa S. (2011). Interleukin-21 and cellular activation concurrently induce potent cytotoxic function and promote antiviral activity in human CD8 T cells. *Human Immunology*.

[30] Pallikkuth S., Parmigiani A., Pahwa S. (2012). The role of interleukin-21 in HIV infection. *Cytokine & Growth Factor Reviews*.

[31] Lee J., Su E. W., Zhu C. (2011). Phosphotyrosine-dependent coupling of Tim-3 to T-cell receptor signaling pathways. *Molecular and Cellular Biology*.

[32] Parry R. V., Chemnitz J. M., Frauwirth K. A. (2005). CTLA-4 and PD-1 receptors inhibit T-cell activation by distinct mechanisms. *Molecular and Cellular Biology*.

[33] Francisco L. M., Sage P. T., Sharpe A. H. (2010). The PD-1 pathway in tolerance and autoimmunity. *Immunological Reviews*.

